# Optimizing rWTC-MBTA Vaccine Formulations, Dosing Regimens, and Cryopreservation Techniques to Enhance Anti-Metastatic Immunotherapy

**DOI:** 10.3390/ijms26031340

**Published:** 2025-02-05

**Authors:** Juan Ye, Herui Wang, Samik Chakraborty, Xueyu Sang, Qingfeng Xue, Mitchell Sun, Yaping Zhang, Ondrej Uher, Karel Pacak, Zhengping Zhuang

**Affiliations:** 1 Neuro-Oncology Branch, Center for Cancer Research, National Cancer Institute, National Institutes of Health, Bethesda, MD 20892, USA; 2 NE1 Inc., New York, NY 10022, USA; samik.chakraborty@ne1inc.com; 3 Section on Medical Neuroendocrinology, Eunice Kennedy Shriver National Institute of Child Health and Human Development, National Institutes of Health, Bethesda, MD 20892, USA

**Keywords:** metastatic cancer, immunotherapy, rWTC-MBTA vaccine, dendritic cells, dosing regimen

## Abstract

Metastatic cancer poses significant clinical challenges, necessitating effective immunotherapies with minimal systemic toxicity. Building on prior research demonstrating the rWTC-MBTA vaccine’s ability to inhibit tumor metastasis and growth, this study focuses on its clinical translation by optimizing vaccine composition, dosing regimens, and freezing techniques. The vaccine formula components included three TLR ligands (LTA, Poly I:C, and Resiquimod) and an anti-CD40 antibody, which were tested in melanoma and triple-negative breast cancer (TNBC) models. The formulations were categorized as rWTC-MBT (Mannan-BAM with LTA, Poly I:C, Resiquimod), rWTC-MBL (LTA), rWTC-MBP (Mannan-BAM with Poly I:C), and rWTC-MBR (Resiquimod). In the melanoma models, all the formulations exhibited efficacy that was comparable to that of the full vaccine, while in the “colder” TNBC models, the formulations with multiple TLR ligands or Resiquimod alone performed the best. Vaccine-induced activation of dendritic cell (DC) subsets, including conventional DCs (cDCs), myeloid DCs (mDCs), and plasmacytoid DCs (pDCs), was accompanied by significant CD80+CD86+ population induction, suggesting robust innate immune stimulation. An initial three-dose schedule followed by booster doses (3-1-1-1 or 3-3-3-3) reduced the metastatic burden effectively. Gradual freezing (DMSO-based preservation) maintained vaccine efficacy, underscoring the importance of intact cell structure. These findings highlight the potential of simplified formulations, optimized dosing, and freezing techniques in developing practical, scalable immunotherapies for metastatic cancers.

## 1. Introduction

In 2022, cancer had a profound global impact, with 20 million new diagnoses and 9.7 million cancer-related deaths [1]. However, this situation is projected to worsen, with 29.9 million new cases expected by 2040 [2,3]. Metastasis, the spread of cancer cells to distant organs, is the primary cause of mortality in these patients and presents a significant challenge in cancer treatment [4,5,6]. In response, precision medicine approaches, including personalized cancer vaccines, are a promising strategy in combating aggressive and metastatic cancer by leveraging the body’s immune system [7,8].

Metastatic cancer cells present significant challenges to therapy by reprogramming themselves to evade immune detection and resist treatment, making them difficult to target [4,5,6]. Patients with surgically removable primary tumors receive systemic therapy to prevent or treat metastasis, while those in later stages primarily focus on treatments extending life [5,6,9,10,11]. Recent advances in immune checkpoint inhibitors, targeted therapies, and antibody–drug conjugates have led to improved survival rates in metastatic cancer [12,13,14].

Immunotherapy has emerged as a leading approach to cancer treatment, with several innovative therapies, such as immune checkpoint inhibitors (ICIs), oncolytic viruses, and chimeric antigen receptor T-cell therapies (CAR-Ts), recently receiving clinical approval [12,13,14,15,16]. Cancer vaccines are a subclass of immunotherapies receiving renewed interest due to their ability to overcome some of the obstacles other immunotherapies are facing, such as antigen escape and durability of the immune response. The primary aim of cancer vaccines is to stimulate a systemic and long-lasting tumor antigen immune response across a broad repertoire of targets [17,18,19,20,21,22,23]. While antitumor efficacy has been demonstrated throughout these treatments, further research is needed to make cancer-targeted immunotherapies more accessible and effective, as high costs and limited availability currently restrict their wider use [7,18,19,21,24,25].

Our research group has successfully developed a personalized cancer vaccine, rWTC-MBTA, which effectively stimulates both innate and adaptive immunity against metastatic cancer cells [26,27,28]. This vaccine consists of irradiated whole tumor cells (rWTC) with Mannan-BAM, toll-like receptor (TLR) agonists (LTA, PolyI:C, and Resiquimod) and an anti-CD40 monoclonal Antibody (mAb). rWTC-MBTA has demonstrated remarkable efficacy in combating colon cancer and metastatic triple-negative breast cancer (TNBC), as well as immunologically “cold” tumors, such as glioblastoma multiforme (GBM), while minimizing systemic toxicity [26,27,28,29]. Mannan, a polysaccharide from *Saccharomyces cerevisiae*, serves as a ligand for phagocytosis when linked to biocompatible anchor for cell membrane (BAM) [30,31]. The Mannan-BAM conjugate attaches to cell membranes through the BAM’s hydrophobic oleyl group, allowing Mannan to interact with pattern recognition receptors (PRRs), which promotes complement activation, opsonization, and phagocytosis of tumor cells [31,32]. Each TLR ligand activates distinct but complementary pathways: LTA from *Bacillus subtilis* stimulates TLR2, triggering inflammatory pathways that increase TNFα secretion [33,34]; Poly(I:C), a synthetic viral dsRNA analogy, activates TLR3, enhancing antigen-presenting cell (APC) activity, inducing apoptosis of tumor cells, and promoting immune supportive macrophage phenotypes [34,35]; and R-848, an imidazoquinolinamine, acts on TLR7/8 (TLR7 in mice), activating innate immunity and inducing a Th1-mediated adaptive immunity [34,36,37]. In addition, the anti-CD40-mAb mimics CD40 ligand interaction on T-helper cells, providing a critical costimulatory signal, further maturing APCs, and bridging innate and adaptive immunity to ensure a robust and long-term memory [38,39,40,41]. Together, these components create a comprehensive, localized immune activation at the injection site, ensuring both immediate and long-term immunity for a potent, targeted antitumor response.

This study investigates critical aspects of the rWTC-MBTA vaccine as it progresses through key stages of preclinical development toward clinical application. The first step in this process involves optimizing the adjuvant combination for each cancer type. Several previous cell and immunotherapy candidates have failed during the transition from preclinical to clinical studies due to improper adjuvant selection [24,29,42]. Several natural and chemically synthesized TLR ligands have been deployed as adjuvants in multiple cancer immunotherapies [29,43,44]. However, certain TLRs were found to have promoted tumor growth and metastasis in specific cancer types [29]. Consequently, it is crucial to carefully select the appropriate tumor types and corresponding TLRs and to explore combination approaches before using TLR-based immunotherapy. Here, we evaluate the efficacy of the rWTC-MBTA variables with fewer TLR ligands as adjuvants in melanoma and TNBC models.

Moreover, despite the promise shown by multiple autologous cell-based cancer therapies in preclinical studies, the impact on patients has been limited [45]. Industry commentaries on unsuccessful products have widely discussed the challenges of successfully bringing novel cell therapies to market, focusing on the inherent complexity of cells, issues with scalability and manufacturing, and regulatory challenges [45]. These factors may influence the transition from preclinical to clinical studies of multi-adjuvant autologous cell-based therapies like rWTC-MBTA. To address these challenges, we are systematically researching the effects of different adjuvants, simplified adjuvant combinations, alternative vaccination regimens, and optimized freezing procedures for cryopreservation. This approach aims to help us overcome regulatory and manufacturing hurdles while identifying feasible adjuvants and dosing strategies for the potential clinical application of the rWTC-MBTA vaccine.

## 2. Results

### 2.1. Comparative Analysis of the Alternative rWTC-MBTA Vaccine Formulations in Melanoma and TNBC Models

Our previous research demonstrated the efficacy of the rWTC-MBTA vaccine in inhibiting tumor metastasis and controlling tumor growth through the enhancement of T-cell-mediated cytotoxicity [26,27,28]. These reports also highlighted its potential as a promising therapeutic approach with minimal systemic toxicity [27,28]. To enhance the clinical translation of the rWTC-MBTA vaccine, we aim to simplify its formulation to ensure easier chemistry, manufacturing, and controls (CMC). Our goal is to compare the efficacy of various vaccine compositions that use only one of the TLR ligands as adjuvants while excluding the anti-CD40 antibody from all alternative formulations. Integrating the anti-CD40 monoclonal antibody as an adjuvant can lead to manufacturing and commercialization issues and autoimmune or other side effects in future clinical applications [41,46,47,48]. We categorized the vaccines based on the present TLR ligands as follows: rWTC-MBT (which includes Mannan-BAM, LTA, Poly I:C, and Resiquimod/R-848), rWTC-MBL (Mannan-BAM plus LTA), rWTC-MBP (Mannan-BAM plus Poly I:C), and rWTC-MBR (Mannan-BAM with Resiquimod/R-848). All the alternative vaccine formulations included irradiated whole tumor cells and were similar to the classical rWTC-MBTA formulation.

**Figure 1:**
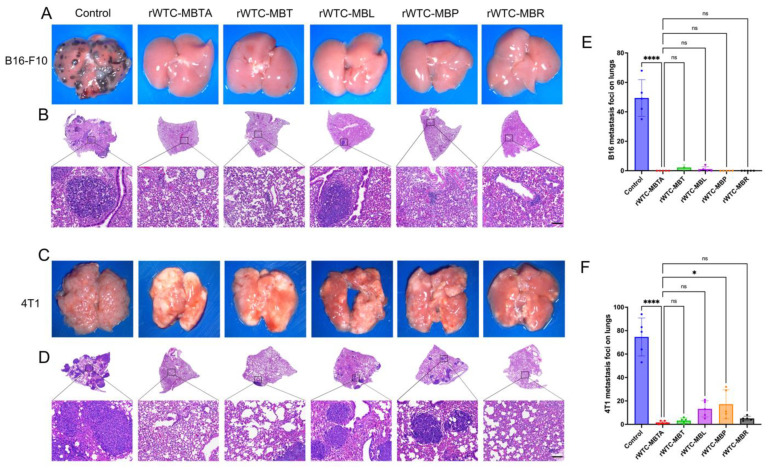
**The anti-metastatic efficacy of vaccines of various compositions in melanoma and breast cancer animal models**. (**A**,**C**) Representative photographs of lung tissues from B16-F10 (**A**) and 4T1 (**C**) metastasis animal models treated with vaccines of different compositions. (**B**,**D**) H&E staining of lung tissues collected on day 45 from B16-F10 (**B**) and 4T1 (**D**) metastatic animal models (scale bar = 100 μm). (**E**,**F**) Quantification of the metastatic loci in the lungs of the mice treated with vaccines of different compositions in the melanoma model (**E**) and the breast animal model (**F**). An ordinary one-way ANOVA test with multiple comparisons assessed the significance of differences. All data are represented as mean ± SD (n ≥ 4). *p*-values are shown for rWTC-MBTA versus vaccines of different compositions. *, *p* < 0.05; ****, *p* < 0.0001; ns, not significant (*p* > 0.05).

Initially, we evaluated the prophylactic efficacy of the various vaccine formulations in a melanoma animal model. Mice were immunized with different vaccine formulations following the regimen established in our previous studies [26,27,28], i.e., three injections weekly for four weeks (3+3+3+3 vaccination scheme). One week after the final vaccination, the mice were challenged with B16-F10 cells via tail vein injection (4 × 10^5^/mouse). As depicted in Figure 1A,B,E and Supplementary Figure S1, the rWTC-MBTA vaccine exhibited significant and comparable anti-metastatic effects with the control. The rWTC-MBT/MBL/MBP/MBR formulations also demonstrated significant anti-metastatic efficacy, though not on the same scale as rWTC-MBTA. The removal of other TLR ligands and the anti-CD40 antibody did not impact the anti-metastatic efficacy.

Since melanoma tumors are considered substantially immune “hot” tumors compared to TNBCs and relatively more straightforward to treat, we conducted additional evaluations of the efficacy of various vaccine formulations in triple-negative breast cancer (TNBC) animal models, known as cold tumors [49,50,51,52,53,54,55,56,57]. In the TNBC animal model, we used six different groups based on the treatment: control, rWTC-MBTA, rWTC-MBT, rWTC-MBL, rWTC-MBP, and rWTC-MBR, with treatments mirroring those used in the melanoma animal model. The results revealed that rWTC-MBT and rWTC-MBR demonstrated comparable anti-metastatic efficacy to the rWTC-MBTA vaccine, though to a slightly lesser extent (Figure 1C,D,F and Supplementary Figure S2). TNBCs are recognized as immune “cold” tumors, presenting challenges for treatment [49,50,55,58]. Consequently, rWTC-MBP and rWTC-MBL did not exhibit the same anti-metastatic efficacy observed in the melanoma animal model. Our findings suggest that MBT may be a viable alternative to MBTA for future clinical applications, as it eliminates the need for an anti-CD40 antibody as an adjuvant. The anti-CD40 monoclonal antibody (mAb) acts like the natural CD40 ligand found on T helper cells and antigen-presenting cells (APCs), including dendritic cells (DCs), B cells, and monocytes. When the anti-CD40 mAb binds to CD40, it activates the APCs and directly stimulates adaptive immune responses [33,41,48]. Our results with alternative formulations of rWTC-MBTA demonstrated the importance of this direct activation of the adaptive immune response through anti-CD40-mAb to curb the tumor metastasis of “cold” tumors like TNBCs. In immunologically “hot” tumors like melanoma, where considerable immune infiltration is prevalent, the triggering of the adaptive immune responses through anti-CD40-mAb may not add any additional benefit, but it can add an advantage to “cold” tumors like TNBCs. In addition, our results also depicted that in “hot” tumors like melanoma, the vaccines prepared with irradiated cells along with just one of the TLR ligands as an adjuvant generated a sufficient anti-metastatic response. However, in “cold” tumors like TNBCs, all three TLR ligands were needed as adjuvants to trigger a substantial anti-metastatic response. These results imply the importance of the presence of different adjuvants in rWTC-MBTA and the variable efficacy in the presence or absence of the anti-CD40 antibody. These results also signaled towards customization of the rWTC-MBTA platform according to the cancer origin, histological type, and characteristics.

### 2.2. Alternative Formulations of the rWTC-MBTA Vaccine Activate the Population of Antigen-Presenting Cells in Local Draining Lymph Nodes of Triple-Negative Breast Cancer and Melanoma Animal Models

Antigen-presenting cells (APCs) are pivotal in connecting innate and adaptive immune responses. Among these, dendritic cells (DCs) are prominent bone marrow-derived cells that emerge from lympho-myeloid hematopoiesis [27,28]. They form a critical interface between innate pathogen sensing and the activation of adaptive immunity [27,28]. This multifaceted task involves a spectrum of mechanisms and responses, which are distributed among three major DC subsets: plasmacytoid DCs (pDCs), conventional DC1s (cDC1s), conventional DC2s (cDC2s), and myeloid DCs (mDCs) [27,28].

**Figure 2:**
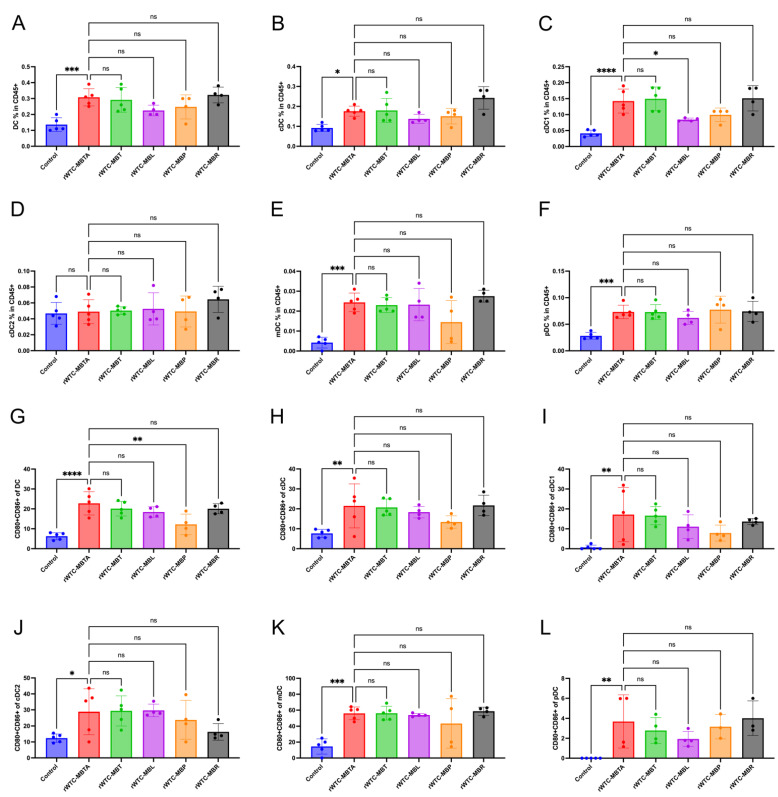
**Vaccines of different compositions enhance antigen presentation by increasing the proportion of dendritic cells in breast cancer animal models**. (**A**–**F**) The percentage of each dendritic cell (DC) subtype (DC, cDC, cDC1, cDC2, mDC, and pDC) in the vaccine-draining lymph nodes and control group 12 h after administration of vaccines of various compositions. (**G**–**L**) CD80+CD86+ percentage of each DC subtype (DC, cDC, cDC1, cDC2, mDC, and pDC) 12 h after administration of vaccines of different compositions. An ordinary one-way ANOVA test with multiple comparisons assessed the significance of differences. All data are represented as mean ± SD (n ≥ 4). *p*-values are shown for rWTC-MBTA versus vaccines of different compositions. *, *p* < 0.05; **, *p* < 0.01; ***, *p* < 0.001; ****, *p* < 0.0001; ns, not significant (*p* > 0.05).

The investigation into their response to various compositions of the rWTC-MBTA vaccine stemmed from their demonstrated efficacy in preventing tumor metastasis and growth. Using a TNBC animal model, we assessed the effect of the alternative formulations of the rWTC-MBTA vaccine on DCs in the draining lymph nodes. We observed an increase in the population of DCs, cDCs, cDC1s, mDCs, and pDCs within the CD45 live cell population across all five groups, including rWTC-MBTA, rWTC-MBT, rWTC-MBL, rWTC-MBP, and rWTC-MBR (Figure 2A–F and Supplementary Figure S3). The significant increase in the mDC population for all vaccine treatments indicates a strong inflammatory response and mobilization of naïve DCs from the bone marrow. Moreover, there was a notable induction of the CD80+CD86+ double-positive cDC1 and mDC populations, particularly within the DC cell subset (Figure 2G–L). We observed similar effects on the DC populations after vaccination with rWTC-MBTA, even in highly immune “cold” tumors like glioblastoma [27]. These results suggest the capability of the alternative formulations of the rWTC-MBTA vaccine to activate innate immune cells like DCs, even with only one TLR ligand as an adjuvant.

### 2.3. The Alternative Formulations of the rWTC-MBTA Vaccine Stimulate Substantial Anti-Metastasis Adaptive Immunity in Both Breast and Melanoma Animal Models

**Figure 3:**
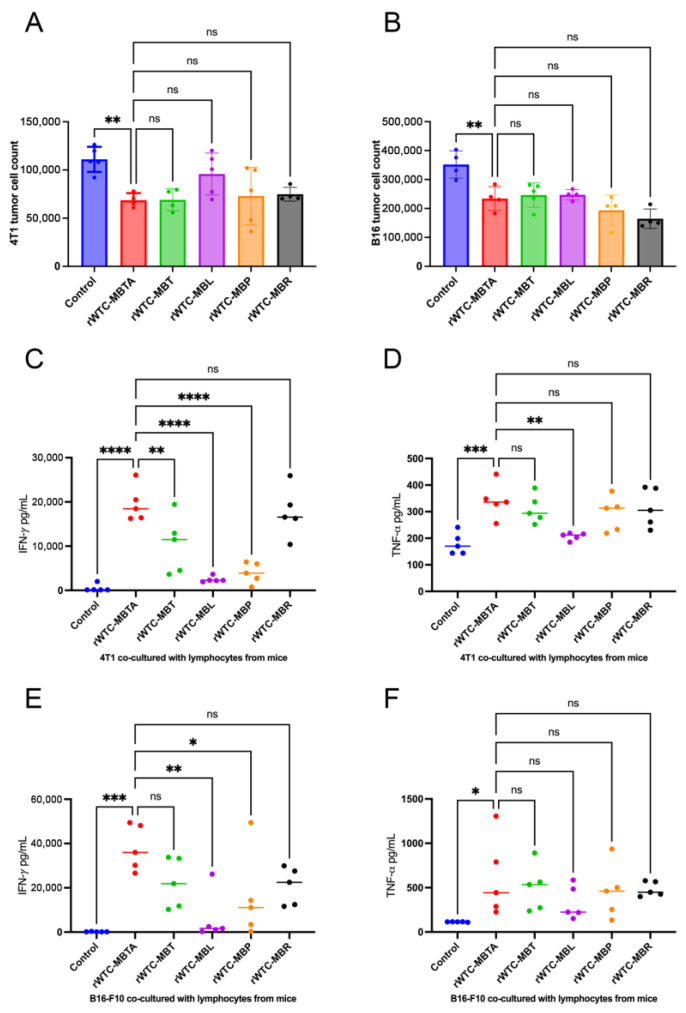
**Vaccines of various compositions stimulate anti-metastasis adaptive immunity in both breast cancer and melanoma animal models.** Lymph nodes were collected and isolated two weeks after the 4T1 tumor challenge by tail vein injection. All the analyses are based on the co-culture 4T1 or B16-F10 tumor cells and the indicated lymphocytes from animals treated with indicated vaccine compositions. (**A**,**B**) Tumor cell counts after co-culturing lymph node cells with 4T1 cells (**A**) and B16-F10 cells (**B**). (**C**–**F**) The concentrations of IFN-*γ* (**C**,**E**) and TNF-*α* (**D**,**F**) in the co-culture supernatants, measured by BD cytometric bead array (CBA). Ordinary one-way ANOVA test with multiple comparisons assessed the significance of differences. The data from panels A and B are represented as mean ± SD (n ≥ 4); the data from (**C**–**F**) are represented as a scatter dot plot with a line at median (n ≥ 4). *p*-values are shown for rWTC-MBTA versus vaccines of different compositions. *, *p* < 0.05; **, *p* < 0.01; ***, *p* < 0.001; ****, *p* < 0.0001; ns, not significant (*p* > 0.05).

Our previous studies with the rWTC-MBTA vaccine delved deeper into the underlying mechanisms and determined that triggering adaptive immunity was responsible for the heightened antitumor efficacy [26,27,28]. In our investigation, we conducted co-culture experiments by harvesting draining lymphoid nodes from mice treated with the alternate vaccine formulations. Subsequently, we isolated immune cells from the lymph nodes and co-cultured them with their relative tumor cells (4T1 and B16-F10). After 48 h, we collected the supernatant for CBA analysis and quantified the tumor cells post-co-culture (Figure 3). Cell counting analysis revealed significantly reduced tumor cell survival after co-culture with lymph node cells from vaccinated mice, compared to the control group (Figure 3A,B). The tumor-specific activation induced in all the vaccine groups was more profound in B16-F10 melanoma cells (Figure 3B) compared to the 4T1 TNBC cells (Figure 3A), and these observations were indicative of the immune “hot” nature of melanoma compared to the TNBC cells. In melanoma, all the alternative formulations of the vaccine exerted comparable activation, whereas, in TNBC cells, the vaccines with only one TLR ligand as adjuvants showed limited efficacy compared to the rWTC-MBTA formulation.

After 48 h, we collected the supernatant and measured pro-inflammatory cytokines like IFN-γ and TNF-α, as in the previous studies [26,27,28]. We observed that MBT (the formulation with all three TLR ligands but no anti-CD40 antibody) could generate a significant adaptive immune response, as indicated by the IFN-γ (Figure 3C,E) and TNF-α levels (Figure 3D,F). However, the vaccine formulations with only LTA or poly-I:C as an adjuvant were not as potent in triggering inflammatory cytokines like IFN-γ and TNF-α. This result indicates the importance of all three TLR ligands in the vaccine to generate potent adaptive immune responses in “cold” tumors.

### 2.4. Fewer Vaccine Doses Were Efficient in Curbing Metastasis in Melanoma Animal Models

To advance the clinical translation of rWTC-MBTA from preclinical to clinical studies, we devised varying injection doses to explore an effective and optimum administration regimen [42,59,60]. We wanted to minimize the dose-limiting toxicity and trauma associated with injection without compromising the efficacy [42]. In addition to the standard dosing protocol (three times per week, five-day gap, for a total of four weeks, abbreviated as 3-3-3-3), we introduced four alternative approaches. These included 3-1-1-1 (three doses in the first week, followed by one dose per week for the subsequent three weeks), 1-1-1-1 (one dose per week for a total of four doses), 3-1-1 (three doses in the first week, followed by one dose per week for the following two weeks), and 3-1 (three doses in the first week, followed by one dose in the subsequent week). In our experimentation using a metastasis animal model with B16-F10 melanoma cells, as in the previous preclinical studies, we initially immunized mice with different injection approaches: control (PBS) (group 1), 3-3-3-3 (group 2), 3-1-1-1 (group 3), 1-1-1-1 (group 4), 3-1-1 (group 5), and 3-1 (group 6), followed by rechallenge with 4 × 10^5^ live B16-F10 tumor cells two weeks after the final vaccination dose. Subsequently, we euthanized the mice two weeks post-challenge and collected lung tissue for metastasis quantification.

**Figure 4:**
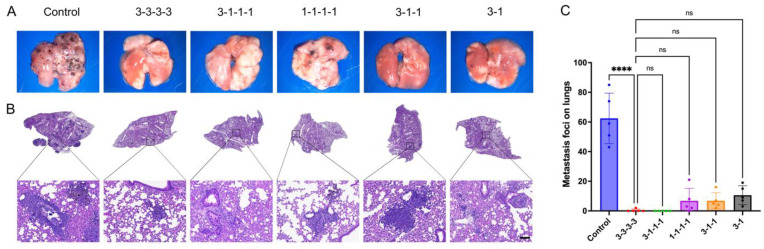
**The anti-metastasis efficacy of different time schedules in melanoma animal models**. (**A**) Representative photographs of lung tissue from B16-F10 metastasis animal models, treated with various vaccine schedules of the rWTC-MBTA formulation. (**B**) H&E staining of lung tissues collected on day 45 from B16-F10 metastatic animal models (scale bar = 100 μm). (**C**) Quantification of metastatic loci in the lungs of mice treated with different vaccine schedules. G1, Control; G2, 3-3-3-3; G3, 3-1-1-1; G4, 1-1-1-1; G5, 3-1-1; G6, 3-1. An ordinary one-way ANOVA test with multiple comparisons assessed the significance of differences between G2 (3-3-3-3) groups and other groups. All data are represented as mean ± SD (n ≥ 4). ns, not significant (*p* > 0.05), ****, *p* < 0.0001.

The results (as depicted in Figure 4A and Supplementary Figure S4) indicated that the 3-3-3-3 group exhibited fewer metastases than the 1-1-1-1, 3-1-1, and 3-1 groups, which is consistent with previous findings. Interestingly, the 3-1-1-1 group demonstrated similar outcomes to the 3-3-3-3 group, suggesting that the 3-1-1-1 injection approach can elicit equivalent antitumor immunity at least. Consequently, we intend to utilize the 3-1-1-1 approach for antitumor interventions in the future. Histological examination via H&E staining further supported these findings, confirming comparable anti-metastatic efficacy between the 3-1-1-1 and 3-3-3-3 approaches (Figure 4B). Quantification of metastatic loci revealed no significant difference between the 3-1-1-1 and 3-3-3-3 approaches, whereas increased metastases were observed in the 1-1-1-1, 3-1-1, and 3-1 protocols (Figure 4C). These results demonstrated the importance of early immune activation by three vaccine doses, followed by single or multiple booster doses for three weeks to maintain the immune responses.

### 2.5. Frozen Vaccines with Freezing Techniques to Preserve the Intact Irradiated Cells Demonstrated Anti-Metastasis Efficacy in Both TNBC and Melanoma Animal Models

Optimizing cell cryopreservation and long-term storage methods is crucial for ensuring the effectiveness of cell-based immunotherapies [61,62]. Freezing and preserving cell therapy products like rWTC-MBTA is critical for maintaining cell viability and guaranteeing the effectiveness of the therapy, and improper preservation can diminish the translational potential of a whole cell vaccine even with promising preclinical results [61,62]. Inadequate preservation techniques are major barriers to the advancement of autologous cell-based immunotherapies and greatly inflate their costs [25,61,62]. In actual clinical scenarios of rWTC-MBTA preparation, patient tumors would be removed through surgery and dissociated into single tumor cells, and the prepared frozen vaccine is then stored for injection [29,63]. Therefore, it is essential to ensure that the frozen rWTC-MBTA vaccine remains viable and effective even after freezing.

To investigate the efficacy of frozen rWTC vaccines, we used different freezing approaches and compared the frozen vaccines against freshly prepared vaccines in both melanoma and breast tumor animal models. We established eight experimental groups, including a negative control (G1) and a positive control (G2). The remaining groups used various methods to prepare vaccines with frozen tumor cells and MBTA: irradiated fresh cultured cells mixed with MBTA and DMSO, gradually frozen, then thawed (G3); irradiated fresh cultured cells mixed with MBTA, quickly frozen, then thawed (G4); quickly frozen tumor lysate, thawed, and mixed with MBTA (G5); fresh cultured cells frozen with DMSO, then thawed and mixed with MBTA as a vaccine (G6); irradiated fresh cultured cells, frozen with DMSO, thawed, and then mixed with MBTA as a vaccine (G7); and double tumor cells (2 × 10^6^) frozen with DMSO, then thawed, irradiated, and mixed with MBTA as a vaccine (G8). The preparation methods for each group are detailed in the Section 4 and figure legends. These involved various approaches to freezing tumor cells and mixing them with MBTA adjuvants, as outlined in the experimental design. For each of the experimental groups, we first immunized mice with the different corresponding vaccine formulations using a 3-3-3-3 injection approach; this was followed by a challenge with tumor cells two weeks post-vaccination via tail vein injection.

**Figure 5:**
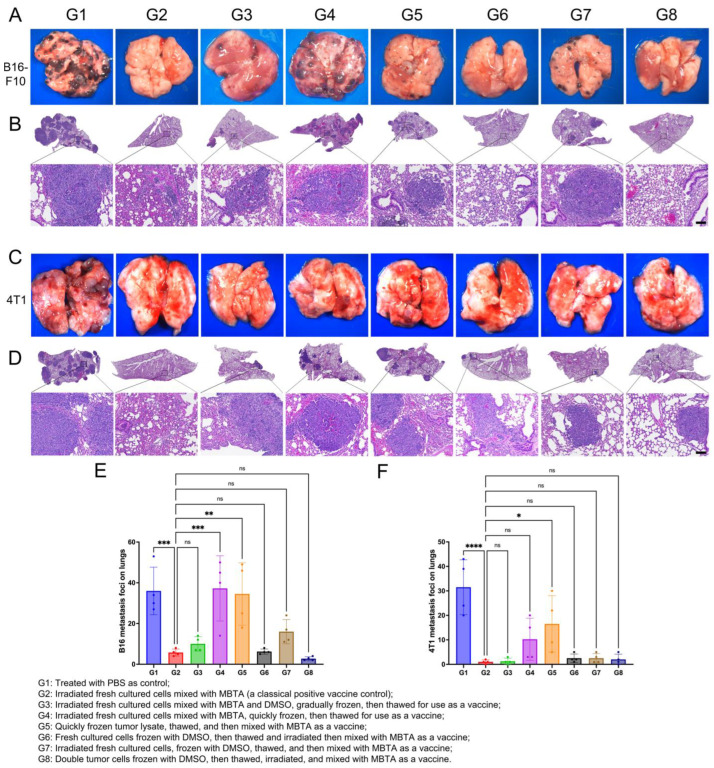
**Anti-metastasis efficacy test of different freezing approaches in both breast cancer and melanoma animal models**. The freezing approaches tested included the following groups: G1: treated with PBS as control; G2: irradiated fresh cultured cells mixed with MBTA (a classical positive vaccine control); G3: irradiated fresh cultured cells mixed with MBTA and DMSO, gradually frozen, then thawed for use as a vaccine; G4: irradiated fresh cultured cells mixed with MBTA, quickly frozen, then thawed for use as a vaccine; G5: quickly frozen tumor lysate, thawed, and then mixed with MBTA as a vaccine; G6: fresh cultured cells frozen with DMSO, thawed and irradiated, and then mixed with MBTA as a vaccine; G7: irradiated fresh cultured cells, frozen with DMSO, thawed, and then mixed with MBTA as a vaccine; G8: double tumor cells frozen with DMSO, then thawed, irradiated, and mixed with MBTA as a vaccine. (**A**,**C**) Representative photographs of lung tissue from B16-F10 (**A**) or 4T1 (**C**) animal models treated with different frozen vaccine strategies. (**B**,**D**) H&E staining of lung tissues collected on day 45 from B16-F10 (**B**) or 4T1 (**D**) metastatic animal models (scale bar = 100 μm). (**E**,**F**) Quantification of the metastatic loci in the lungs of mice using different frozen strategy vaccines in melanoma (**E**) and breast cancer animal models (**F**). Statistical significance was assessed using the ordinary one-way ANOVA test with multiple comparisons. All data are represented as mean ± SD (n ≥ 4). *p*-values are shown for rWTC-MBTA versus vaccines of different compositions. *, *p* < 0.05; **, *p* < 0.01; ***, *p* < 0.001; ****, *p* < 0.0001; ns, not significant (*p* > 0.05).

In the melanoma animal model, the results revealed that the vaccines prepared according to groups G4 and G5 failed to inhibit metastases compared to G2, G3, G6, G7, and G8. This suggests that once the tumor cell integrity is compromised, it becomes less effective for vaccine production due to the lack of tumor antigen (refer to Figure 5A,B,E and Supplementary Figure S5). Notably, G3, G6, and G8 exhibited anti-metastatic efficacy that was comparable to our freshly prepared rWTC-MBTA vaccine (G2), highlighting the importance of the gradual freezing technique and the preservation of cell structures for effective vaccine performance. Although metastasis was somewhat higher in G7, there were no significant differences between G7 and G2, indicating that G7 also retains comparable anti-metastatic efficacy to G2 and reinforcing the critical role of maintaining cell integrity in developing effective tumor cell-based vaccines.

Similar results were observed in the breast tumor animal model, where the integrity of the frozen tumor cells significantly impacted the vaccine’s efficacy (Figure 5C,D,F and Supplementary Figure S5. In this model, G3, G6, G7, and G8 demonstrated anti-metastatic effects comparable to G2, with G7 showing slightly enhanced efficacy in breast tumors compared to melanoma. This enhanced effect in breast tumors may reflect a greater sensitivity of these tumors to immune stimulation from partially preserved antigens, emphasizing the need for optimized cryopreservation protocols to maintain the reliability and potency of cell-based vaccines across different tumor types.

## 3. Discussion

Previous studies from our collaborating research group on intra-tumoral MBTA injections showed strong antitumor effects [64,65]. Earlier research combined Mannan-BAM with TLR agonists to trigger immunity in colon cancer, TNBC, and GBM models, leading to significant tumor reduction and, in some cases, complete remission in mice [26,27,28]. In a colon carcinoma mouse model, the rWTC-MBTA vaccine (irradiated tumor cells with MBTA) significantly reduced tumor size and improved survival [26]. Recent studies showed that this vaccine also inhibited tumor growth and metastasis in breast cancer and melanoma models, with effects dependent on T-cell activation and immune memory [27,28]. In the latest article about rWTC-MBTA, we explored the therapeutic efficacy of the rWTC-MBTA vaccine in murine GBM models, showing that it effectively activated DC subsets, induced strong tumor-specific adaptive immune responses against GBM, achieved remission, and established long-term immune memory [27]. Given this preclinical success, further clinical investigation of rWTC-MBTA is warranted as potential cancer immunotherapy. However, in this research study, we are focusing more on the translational aspects, like composition and vaccination regimen without compromising efficacy, as the immune response mechanisms are already established.

Autologous tumor cell-based vaccines like rWTC-MBTA use whole irradiated tumor cells, which allows them to be prepared more rapidly compared to vaccines that rely on tumor-specific antigens. This approach bypasses the need for sequencing, predicting, and verifying individual antigens. An additional benefit of rWTC-MBTA is its straightforward preparation process, which bypasses clinical-grade chemosynthesis. This could revolutionize the autologous tumor cell-based vaccine scenario by reducing the time from surgery to vaccination [26,27,28,29]. In prospective clinical studies, the personalized strategy of rWTC-MBTA will utilize irradiated whole tumor cells isolated from surgically removed patient tumors to create tumor antigen and neoantigen pools. We have incorporated Mannan-BAM as a PAMP to enhance the immunogenicity [20,29,63]. Mannan-BAM activates the lectin complement pathway, leading to iC3b opsonization of tumor cells. In this process, the PAMP chemotactically attracts phagocytic cells, dendritic cells, macrophages, and other antigen-presenting cells (APCs), leading to enhanced opsonization and phagocytosis of intact cancer cells by amplifying neoantigen presentation manyfold [20,29,63]. PAMPs are evolutionarily conserved immune modulators, guaranteeing an immune response irrespective of the application [20]. In addition, the TLR ligands and anti-CD40 antibody in the rWTC-MBTA are used as adjuvants to amplify the tumor-specific immune response [29,63]. Except for LTA, all other adjuvants are used in several clinical trials, including cancer immunotherapies [29]. The regulatory agencies mandate comprehensive information about adjuvants early in the vaccine development process, particularly if the adjuvants exhibit independent clinical activity [66]. Multi-adjuvant therapeutics, like rWTC-MBTA, require extensive regulatory oversight due to their high safety/toxicity concerns [66]. There are some published preclinical studies regarding the toxicity of Resiquimod and poly-IC in mouse models [67,68,69]. Those studies involve preclinical experimental setups (choice of mouse models, significantly higher dosages, and dosing regimens) that are entirely different from rWTC-MBTA [26,27,28,67,68,69]. However, in our previous studies, rWTC-MBTA demonstrated zero toxicity, including no hepatotoxicity or nephrotoxicity [27,28]. Several clinical and preclinical immunotherapeutic approaches showed negligible side effects and toxicity, including TLR ligands, Resiquimod, and poly-IC as adjuvants [29]. With increasing knowledge of TLRs and their signaling pathways, natural and synthetic TLR agonists are integrated into cancer immunotherapy [29]. However, early clinical challenges have arisen due to some TLRs promoting tumor growth and metastasis in certain cancers. Therefore, selecting the right tumor type and TLRs and using combinatorial approaches for effective TLR-based immunotherapy is crucial. Given the flexibility of the rWTC-MBTA platform, one can remove TLR ligands based on the target cancer type to reduce the chances of tumor progression and metastasis. The omission of the anti-CD40 antibody from the rWTC-MBTA composition was a translational modification to reduce potential regulatory obstacles and the high cost of licensing the antibody technology.

Determining the optimum vaccination regimen for any vaccine is crucial for its utmost efficacy. A lower number of vaccinations may result in an inadequate immune response due to a lack of enough antigens. Conversely, higher doses of vaccine could lead to dose-limiting toxicity (DLT), cytokine release syndrome (CRS), or an excessive innate immune response leading to adverse side effects [21,42]. Yet, the variability in immune response relationships across different antigens or epitopes in multi-adjuvant personalized autologous tumor cell-based vaccines makes it challenging to deduce the precise relations between the individual components and the resulting anticancer immune and clinical outcomes [42]. Furthermore, we intend to use resected patient tumors in future clinical studies to isolate the autologous tumor cells for vaccine preparation. The number of cells can vary from patient to patient, which in turn affects the available number of doses. Therefore, we wanted to estimate a minimum number of vaccine doses to prevent tumors. We used the mouse models to understand the minimum number of doses of rWTC-MBTA required to generate an adequate antitumor immune response. Different vaccination regimes result in diverse T-cell responses, and the antigen dose is a crucial determinant of the vaccine efficacy. The right amount of tumor antigens can influence the early antitumor cytotoxic T-cell response and can generate adequate memory T-cells [59]. Extremely low levels of antigens can generate T-cells with higher avidity, whereas elevated antigen levels could result in diminished T-cell functionality [59]. Several animal and human studies demonstrated that lower amounts of vaccine antigens in doses were found to elicit better T-cell responses and elevated immune effectors and displayed improved efficacy against future incursions [59]. Our investigation of various vaccination regimens yielded notable findings. The 3+3+3+3 injection regime demonstrated maximum efficacy in preventing metastasis in both the melanoma and TNBC models. The 3+1+1+1 vaccination can also prevent the metastasis of cancer cells to the lungs to an extent, but the effect was more profound on melanoma than TNBC. This can be explained by the fact that melanoma has a more immune “hot” nature than TNBC, making them responsive to immunotherapy. These results elucidate the importance of determining the appropriate vaccination regime based on the immune profile of the tumor, and these can improve the efficacy and the postoperative quality of life.

In addition to the composition and the dosing regimen, the last manufacturing parameter of rWTC-MBTA we examined was the freezing of the vaccine for future use. Preservation by freezing, especially by cryofreezing the product for future injections, is an essential step for cell therapy [25,61,62]. Proper freezing of the vaccine can maintain the stability of the adjuvants as well as the integrity of the cancer cells. Frozen vaccines must remain readily available over time without degradation of the contents [25,61,62]. Cooling approaches, such as cooling media and the freezing process, can damage the vaccine, thus compromising its efficacy [25,61,62]. We evaluated the efficacy of different freezing procedures of rWTC-MBTA to determine the best approach to freeze and store the vaccine. We found that a slow freezing approach retained almost similar efficacy compared to the freshly prepared vaccine from the cultured cells. The slow freezing method likely reduced irradiated cell damage and, consequently, preserved tumor antigen quality [25,61,62].

Overall, the study provides valuable insights into the optimization of vaccine formulations and dosing strategies for effective anti-metastatic immunotherapy. Identifying promising alternative vaccine candidates with different adjuvant combinations paves the way for further preclinical and clinical investigations. However, we acknowledge the inherent challenges in translating findings from preclinical models to humans. The genetic and phenotypic heterogeneity of human tumors, as well as differences in vaccine preparation, schedule, and composition, pose significant barriers to direct application. To address these limitations, we included a discussion on the need for further translational studies to validate the efficacy and safety of these vaccine formulations in human clinical trials. Personalized vaccine formulations and optimized clinical protocols that account for the variability in patient-specific tumor characteristics will be critical to developing novel immunotherapeutic approaches for metastatic cancer treatment.

## 4. Methods

### 4.1. Materials and Cell Lines

Mannan (*Saccharomyces cerevisiae*), polyinosinic-polycytidylic acid sodium salt, and lipoteichoic acid (*Bacillus subtilis*) were purchased from Sigma-Aldrich (St. Louis, MO, USA). Biocompatibility anchors for cell membranes (BAMs) were obtained from NOF America (White Plains, NY, USA), and Resiquimod (R-848) was sourced from Tocris Bioscience (Minneapolis, MN, USA). Anti-mouse CD40 (clone FGK4.5/GFK45) was supplied by Bi-oXCell (West Lebanon, NH, USA). Antibodies for flow cytometry, including anti-CD45, anti-CD3e, anti-Ter119, anti-NK1.1, anti-CD11b, anti-CD11c, anti-CD8a, and anti-F4/80, were purchased from BD Biosciences (Franklin Lakes, NJ, USA), while anti-CD80 and anti-CD86, along with CBA kits for IFN-γ, TNF-α, and IL-6 detection, were procured from Bioligand (San Diego, CA, USA).

B16-F10 melanoma and 4T1 mouse mammary tumor cells were obtained from ATCC and cultured in DMEM and RPMI 1640 medium, respectively, supplemented with 10% fetal bovine serum (FBS) and 1% penicillin/streptomycin (PS). All media, FBS, and antibiotics were sourced from Gibco (Gaithersburg, MD, USA).

### 4.2. Animal

Healthy female mice of BALB/c and C57BL/6 (around 6–8 weeks old) were purchased from Charles River Laboratories (Wilmington, MA, USA). All animal experiments were carefully reviewed and approved by the NCI Animal Care and Use Committee (ACUC) to ensure compliance with federal regulations and ethical standards. These procedures were carried out by NCI-CCR staff as part of the intramural NIH ACU program, which is fully accredited by AAALAC International.

### 4.3. rWTC-MBTA Vaccine Preparation

The procedure consists of two steps. In the first step, we synthesized MBTA-BAM following a previously reported method [26]. Aminated Mannan was prepared using a reductive amination approach [70]. Specifically, Mannan was dissolved in ammonium acetate (300 mg/mL) and subjected to reduction with 0.2 M sodium cyanide at pH 7.5 and 50 °C for 5 days. The solution was then dialyzed against PBS at 4 °C overnight using MWCO 3500 dialysis tubing (Serva, Heidelberg, Germany). For the other optimized vaccine preparation, one component was reduced in each vaccine formulation. According to Kato et al., BAM binds to the amino group of mannoprotein at pH 7.3 [30]. The N-hydroxysuccinimide (NHS) group of BAMs was allowed to react with the amino group of Mannan at room temperature for one hour. The resulting conjugate was dialyzed overnight against PBS at 4 °C in MWCO 3500 dialysis tubing (Serva, Heidelberg, Germany), yielding Mannan-BAM (MB). The MBTA solution was prepared by combing 50 μL of 0.2 mM Mannan-BAM solution with 25 μg R-848 (hydrochloric acid form), 25 μg poly(I:C), 25 μg LTA, and 1 μg anti-CD40. The MBTA formulation contains Mannan-BAM, LTA, poly (I:C), and R-848; the MBT formulation includes Mannan-BAM, LTA, Poly I:C, and R-848; the MBL formulation contains Mannan-BAM and LTA; the MBP consists of Mannan-BMA and poly(I:C); the MBR includes Mannan-BAM and R-848. All the resulting solutions were filtered using a 0.22 μm filter and stored at −20 °C until use.

The next step involves irradiating whole tumor cells and combining them with different components. The whole tumor cells (1 × 10^6^ tumor cells suspended in 50 μL PBS for each mouse dose) were exposed to 100 Gy using a 137Cs MARK I model irradiator (JL Shepherd & Associates, San Fernando, CA, USA). After irradiation, the whole irradiated tumor cells (50 μL, per mouse) were incubated with various formulations of MBTA, MBT, MBL, MBP, or MBR (50 μL/mouse) for 0.5 to 1 h at room temperature. Following this, the different vaccine formulations (100 μL per mouse) were injected subcutaneously into the right flank of the treated animals according to the designated treatment schedule.

### 4.4. For the Various Frozen Vaccine Approaches

Tumor cells or lysates were prepared using different freezing methods. Some tumor cells were frozen in 10% DMSO, while others were frozen without DMSO. Tumor lysates were also frozen using either rapid freezing or programmed cooling methods. Prior to injection, all the frozen cells or lysates were thawed and mixed with MBTA to create the vaccine. The experimental groups were as follows: G1: PBS-treated control; G2: irradiated fresh cultured tumor cells mixed with MBTA, serving as a classical positive control; G3: irradiated fresh cultured tumor cells mixed with MBTA and DMSO, gradually frozen, then thawed for use as a vaccine; G4: irradiated fresh cultured tumor cells mixed with MBTA, quickly frozen, then thawed for use as a vaccine; G5: quickly frozen tumor lysate, thawed, and mixed with MBTA for use as a vaccine; G6: fresh cultured tumor cells frozen with DMSO, then thawed and mixed with MBTA for use as a vaccine; G7: irradiated fresh cultured tumor cells frozen with DMSO, then thawed and mixed with MBTA for use as a vaccine; and G8: double tumor cells frozen with DMSO, then thawed, irradiated, and mixed with MBTA for use as a vaccine.

### 4.5. T-Cell Activation Assay

To evaluate the T-cell activation immune response, we conducted a co-culture assay. Mice were immunized with rWTC-MBTA, rWTC-MBT, rWTC-MBL, rWTC-MBP, rWTC-MBR, or PBS. Immunizations were administered daily for four weeks, with three injections per week. One week after the final immunization, mice were inoculated with either 2 × 10^5^ 4T1 cells or 4 × 10^5^ B16-F10 cells. Two weeks post-inoculation, lymph nodes were harvested, manually ground, and filtered through a 70 μm MACS SmartStrainer (Miltenyi Biotec, Inc., Gaithersbury, MD, USA) for tissue dissociation. Red blood cells were lysed using ACK lysis buffer, followed by a wash with PBS and centrifugation at 500× *g* for 10 min. Based on a previous study [71,72], the isolated lymph node cells were co-cultured with 4T1 or B16-F10 tumor cells at a 10:1 ratio. After 48 h of co-culture, the supernatant was collected for cytokine analysis (IFN-γ and IFN-α) using a CBA assay (BD Biosciences, North Brunswick Township, NJ, USA). Co-cultured cells were stained with anti-CD4, anti-CD8, anti-CD45, and anti-TCR-β antibodies for T-cell cytotoxicity analysis. Tumor cell counts were determined using counting beads (Life Technologies | Invitrogen, Waltham, MA, USA) for flow cytometry analysis; 10 μL of counting beads (Life Technologies | Invitrogen, Waltham, MA, USA) was added to each well of co-culture samples, and the cells were stained with a live dye and an anti-CD45 antibody. The tumor cells were identified as live/CD45- cells because more than 95% of the splenocytes were CD45+, and the remaining CD45- splenocytes could be distinguished from tumor cells by size (forward scatter). Tumor cell counts were determined by counting the number of tumor cells and beads among the gated live cells. Since a fixed number of beads (10,000) was added to each sample, the number of surviving tumor cells was calculated as follows: (counted tumor cell number)/(counted bead number) × 10,000 (total beads number).

### 4.6. Dendritic Cell Profiling Following Immunization with Differently Formulated Vaccines

To investigate the transient immune response, we evaluated the changes in proportions of antigen-presenting cells following treatment with differently formulated vaccines; we immunized groups of BALB/c mice with r4T1-MBTA, r4T1-MBT, r4T1-MBL, r4T1-MBP, r4T1-MBR, or PBS. Twelve hours later, lymph nodes were collected from the immunized mice and dissociated into a single-cell suspension, which was stained with anti-CD45, dump channel (anti-CD3/CD19/Ter119/NK1.1), anti-CD11c, CD11b, Ly6C, MHCII, anti-CD80, and anti-CD86 antibodies. CD45+DUMP-MHCII+CD11c+ antibodies were gated as dendritic cells (DC); anti-CD80+anti-CD86+ dendritic cells were gated as mature dendritic cells; CD45+DUMP-CD11b-Ly6C+ was gated as pDC; CD45+DUMP-CD11b+Ly6C+ was gated as mDC; CD45+DUMP-Ly6C- was gated as cDC; CD45+DUMP-CD11b- was gated as cDC1; CD45+DUMP-CD11b+ was gated as cDC2. Flow cytometry was performed using a FACSymphony (BD Biosciences).

## 5. Statistical Analysis

The concentrations of cytokines (IFN-r and TNF-a) were presented as a scatter dot plot with a line at the median (n ≥ 4). All other data were presented as mean ± standard deviation (SD) (n ≥ 4). An ordinary one-way ANOVA test with Dunnett’s multiple comparison tests was performed. Statistical analyses and the formation of figures were conducted using GraphPad Prism 8.4.0 (GraphPad Software, La Jolla, CA, USA). *p*-values < 0.05 were considered statistically significant.

## 6. Conclusions

This study demonstrates that alternative formulations of the rWTC-MBTA vaccine, even with single TLR ligands, maintain significant anti-metastatic efficacy in melanoma and triple-negative breast cancer (TNBC) models. This offers the potential for creating simplified yet effective vaccine compositions.

The research also highlights that optimized dosing regimens, which involve early intensive administration followed by booster doses, are crucial for enhancing anti-metastatic responses while minimizing systemic toxicity. Additionally, the use of gradual freezing methods has preserved the integrity and efficacy of frozen vaccine preparations, enabling practical storage solutions without compromising therapeutic potential.

Overall, these findings enhance our understanding of the composition, dosing, and preservation strategies for the rWTC-MBTA vaccine, presenting streamlined approaches for clinical application.

## Data Availability

Data supporting the results of this study can be found in the article and its Supplementary Materials. The original data and reagents used in this study are available from the corresponding authors upon request.

## Supplementary Materials

The following supporting information can be downloaded at: https://www.mdpi.com/article/10.3390/ijms26031340/s1.
