# Supplementary material for: Optimizing rWTC-MBTA Vaccine Formulations, Dosing Regimens, and Cryopreservation Techniques to Enhance Anti-Metastatic Immunotherapy
Source: Int J Mol Sci. 2025 Feb 5;26(3):1340. doi: 10.3390/ijms26031340 (PMC11818183; doi:10.3390/ijms26031340)
Supplement: Supplementary file 1 [file ijms-26-01340-s001.zip › ijms-3263207-supplementary.pdf]

Optimizing rWTC-MBTA Vaccine Formulations, Dosing Regimens, and Cryopreservation Techniques to Enhance Anti-metastatic Immunotherapy

Juan Ye[1#*], Herui Wang[1#], Samik Chakraborty[2], Xueyu Sang[1], Qingfeng Xue[1], Mitchell Sun[1], Yaping Zhang[1], Ondrej Uher[3], Karel Pacak[3], Zhengping Zhuang*[1]

1, Neuro-Oncology Branch, Center for Cancer Research, National Cancer Institute, National Institutes of Health, Bethesda, Maryland, USA.

2, NE1 Inc., New York, NY, USA.

3, Section on Medical Neuroendocrinology, *Eunice Kennedy Shriver* National Institute of Child Health and Human Development, National Institutes of Health, Bethesda, Maryland, USA

# These authors contributed equally

*Correspondence:

Juan Ye, Ph.D.

Postdoctoral Fellow

Neuro-Oncology Branch

National Cancer Institute

National Institutes of Health

37 Convent Drive,

Building 37, Room 1014A

Bethesda, MD 20892

juan.ye@nih.gov

Zhengping Zhuang, M.D., Ph.D.

Senior Investigator

Neuro-Oncology Branch

National Cancer Institute

Center for Cancer Research

National Institutes of Health

Building 37, Room 1000

37 Convent Dr.

Bethesda, MD 20892

zhengping.zhuang@nih.gov

Supplementary figure legend

Supplementary figure 1

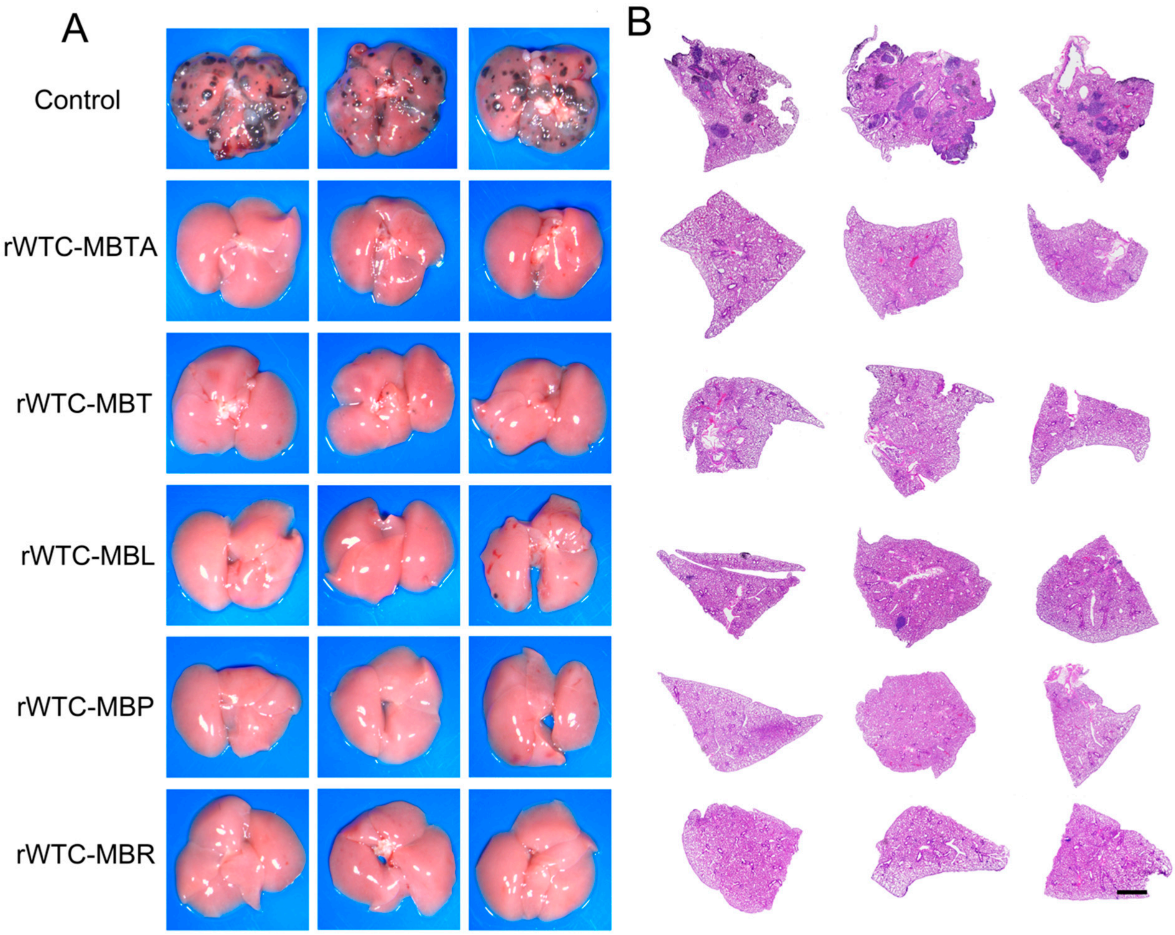

Figure S1. **Photographs (A) and H&E staining of lung sections (B) of the B16-F10 animal models treated with different vaccine compositions (including MBTA, MBT, MBL, MBP, and MBR). Scale bar=1mm.**

Supplementary figure 2

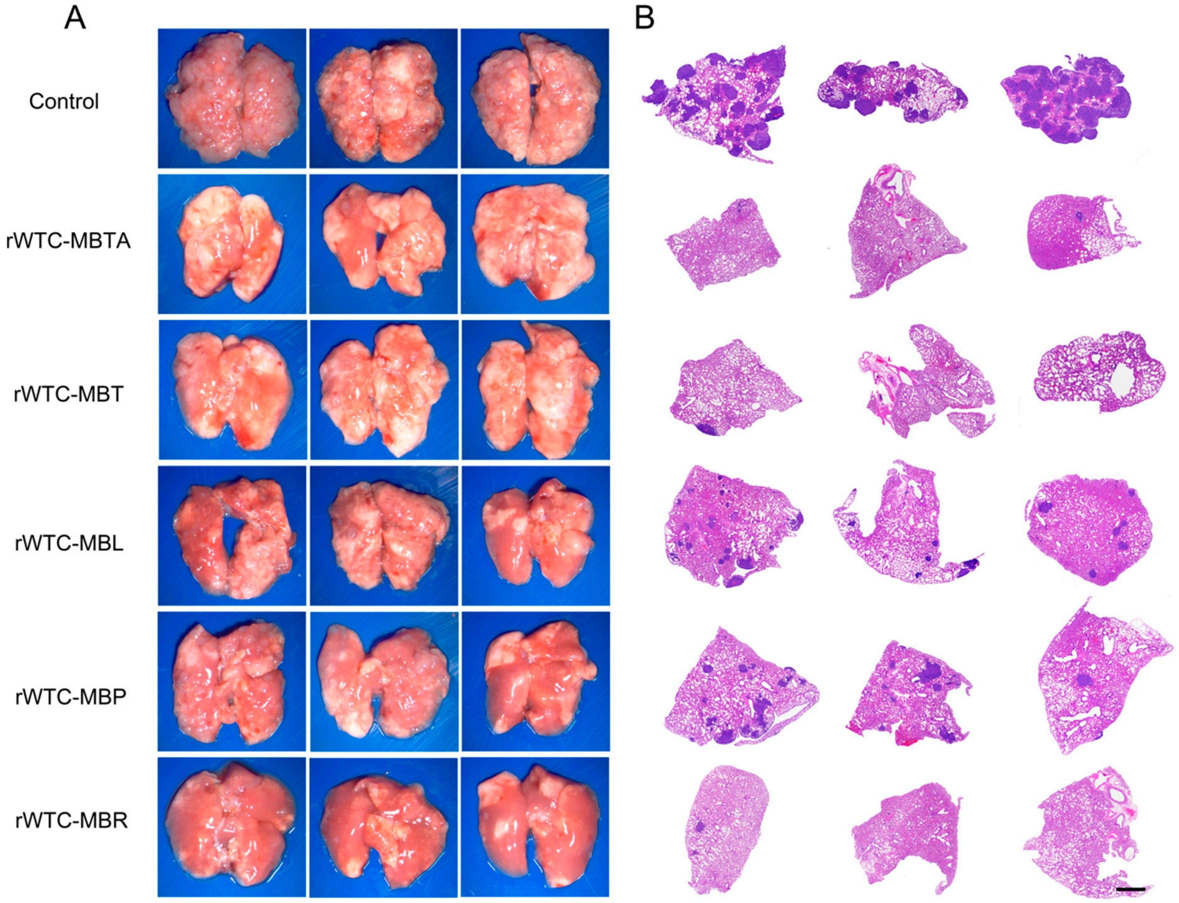

Figure S2. **Photographs (A) and H&E staining of lung sections (B) of the 4T1 animal models treated with different vaccine compositions (including MBTA, MBT, MBL, MBP, and MBR). Scale bar=1mm.**

Supplementary figure 3

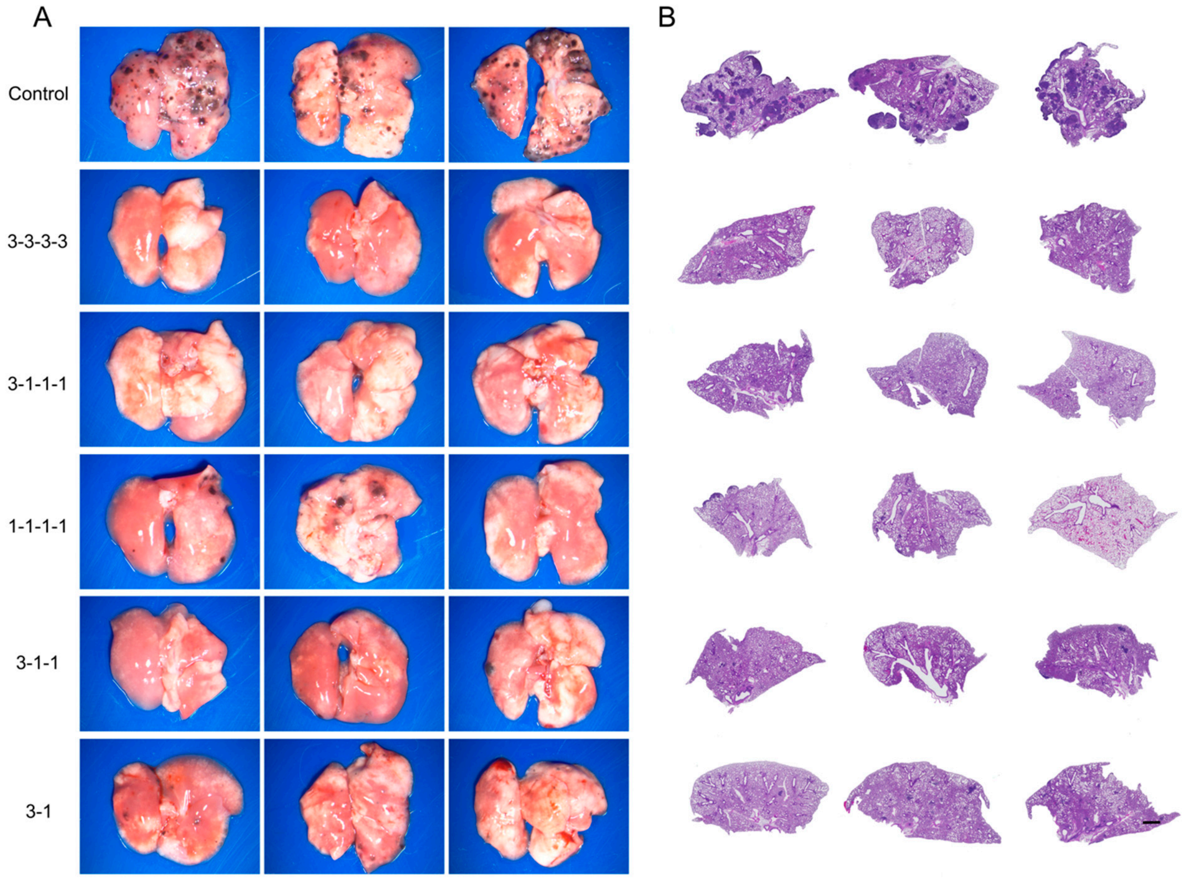

Figure S3. **Photographs (A) and H&E staining of lung sections (B) of the 4T1 animal models treated with different vaccine schedule strategies (including 3-3-3-3, 3-1-1-1, 1-1-1-1, 3-1-1 and 3-1). Scale bar=1mm.**

Supplementary figure 4

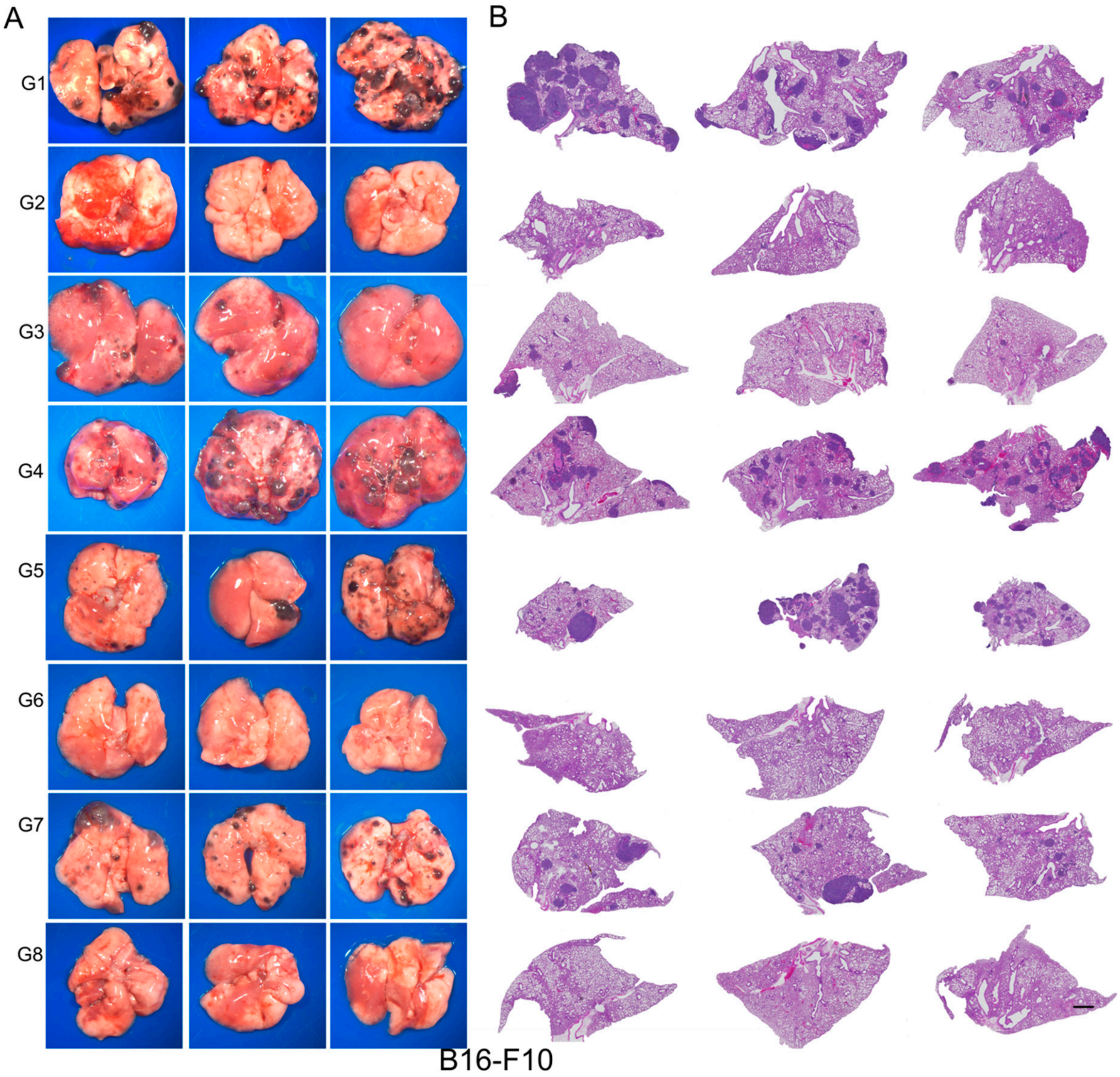

Figure S4. **Photographs (A) and H&E staining of lung sections (B) of the melanoma animal models treated with various freezing strategies,** which included the following groups: G1: Treated with PBS as control; G2: Irradiated fresh cultured cells mixed with MBTA as vaccine (a classical positive control); G3: Irradiated fresh cultured cells mixed with MBTA and DMSO, gradually frozen, then thawed for use as a vaccine; G4: Irradiated fresh cultured cells mixed with MBTA, quickly frozen, then thawed for use as a vaccine; G5: Quickly frozen tumor lysate, thawed, and then mixed with MBTA as a vaccine; G6: Fresh cultured cells frozen with DMSO, thawed and irritated, then mixed with MBTA as a vaccine; G7: Irradiated fresh cultured cells, frozen with DMSO, thawed, and then mixed with MBTA as a vaccine; G8: Double tumor cells frozen with DMSO, then thawed, irradiated, and mixed with MBTA as a vaccine. Scale bar=1mm**.**

Supplementary figure 5

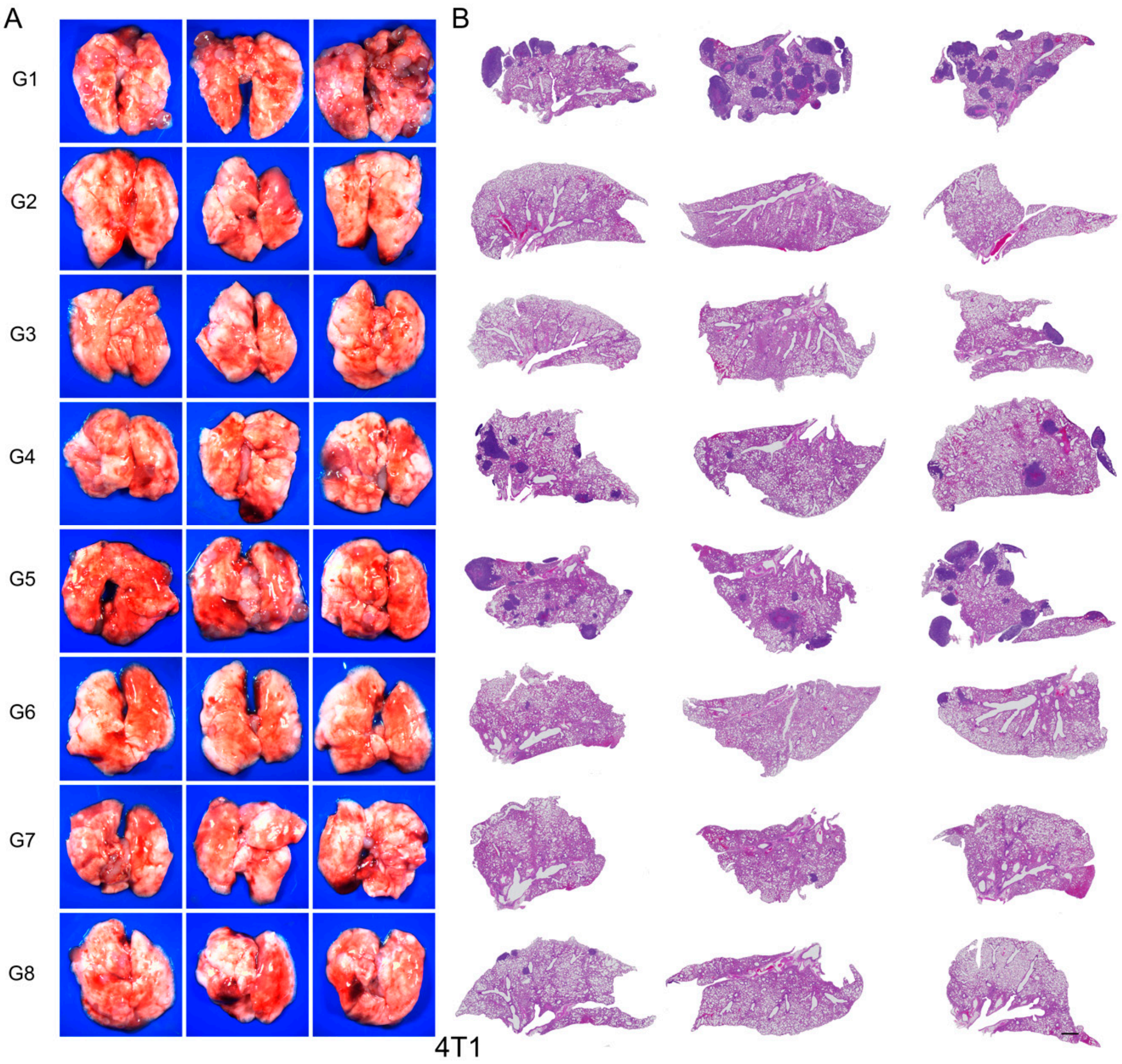

Figure S5. **Photographs (A) and H&E staining of lung sections (B) of the 4T1 animal models treated with various freezing strategies,** which included the following groups: G1: Treated with PBS as control; G2: Irradiated fresh cultured cells mixed with MBTA as vaccine (a classical positive control); G3: Irradiated fresh cultured cells mixed with MBTA and DMSO, gradually frozen, then thawed for use as a vaccine; G4: Irradiated fresh cultured cells mixed with MBTA, quickly frozen, then thawed for use as a vaccine; G5: Quickly frozen tumor lysate, thawed, and then mixed with MBTA as a vaccine; G6: Fresh cultured cells frozen with DMSO, thawed and irradiated, then mixed with MBTA as a vaccine; G7: Irradiated fresh cultured cells, frozen with DMSO, thawed, and then mixed with MBTA as a vaccine; G8: Double tumor cells frozen with DMSO, then thawed, irradiated, and mixed with MBTA as a vaccine. Scale bar=1mm.
